# *PILRA* polymorphism modifies the effect of *APOE*4 and *GM*17 on Alzheimer’s disease risk

**DOI:** 10.1038/s41598-022-17058-6

**Published:** 2022-08-02

**Authors:** Karin Lopatko Lindman, Caroline Jonsson, Bodil Weidung, Jan Olsson, Janardan P. Pandey, Dmitry Prokopenko, Rudolph E. Tanzi, Göran Hallmans, Sture Eriksson, Fredrik Elgh, Hugo Lövheim

**Affiliations:** 1 Department of Community Medicine and Rehabilitation, Geriatric Medicine, Umeå University, 901 85 Umeå, Sweden; 2 Department of Public Health and Caring Sciences, Geriatric Medicine, Uppsala University, Uppsala, Sweden; 3 Department of Clinical Microbiology, Umeå University, Umeå, Sweden; 4 Department of Microbiology and Immunology, Medical University of South Carolina, Charleston, USA; 5 Genetics and Aging Unit, Department of Neurology, McCance Center for Brain Health, Massachusetts General Hospital, Boston, MA USA; 6 Harvard Medical School, Boston, MA USA; 7 Department of Public Health and Clinical Medicine, Umeå University, Umeå, Sweden; 8 Wallenberg Centre for Molecular Medicine (WCMM), Umeå University, Umeå, Sweden

**Keywords:** Genetics, Diseases, Neurological disorders

## Abstract

*PILRA* (rs1859788 A > G) has been suggested to be a protective variant for Alzheimer’s disease (AD) and is an entry co-receptor for herpes simplex virus-1. We conducted a nested case–control study of 360 1:1-matched AD subjects. Interactions between the *PILRA*-A allele*, APOE* risk variants (ε3/ε4 or ε4/ε4) and *GM*17 for AD risk were modelled. The associations were cross-validated using two independent whole-genome sequencing datasets. We found negative interactions between *PILRA*-A and *GM*17 (OR 0.72, 95% CI 0.52–1.00) and between *PILRA*-A and *APOE* risk variants (OR 0.56, 95% CI 0.32–0.98) in the discovery dataset. In the replication cohort, a joint effect of *PILRA* and *PILRA* × *GM* 17/17 was observed for the risk of developing AD (*p* .02). Here, we report a negative effect modification by *PILRA* on *APOE* and *GM*17 high-risk variants for future AD risk in two independent datasets. This highlights the complex genetics of AD.

## Introduction

The underlying cause of Alzheimer´s disease (AD) is considered to involve both genetic and environmental factors^1^. The major genetic risk allele for late-onset AD is the ε4 variant of the apolipoprotein E gene (*APOE*) on chromosome 19^2^. Large genome-wide association studies (GWAS) have discovered several other risk loci for AD^3,4^, many of which are also associated with immune dysfunction in the central nervous system^5^. Using a candidate gene approach, a new potential risk variant for AD was identified in the immunoglobulin heavy chain G (*IGHG*) genes on chromosome 14^6^. The risk allele of *IGHG* encodes the immunoglobulin (Ig) Ƴ marker (*GM*) 17 allotype, and homozygosity for *GM*17 was independently associated with a fourfold increased risk of AD. Interestingly, both *APOE* and *GM*17 might affect host susceptibility to herpes simplex virus 1 (HSV-1) infections^6–10^. Another gene implicated in AD predisposition is *PILRA* located on chromosome 7^11–14^.

*PILRA* encodes the protein paired immunoglobulin-like type 2 receptor alpha (PILRA), an inhibitory surface receptor expressed by myeloid cells and other tissues including the nervous system^15^, which appears to regulate immune cells and inflammation^16–18^. Also, PILRA plays an important role in the life cycle of HSV-1, acting as an entry co-receptor for HSV-1 through the binding of viral glycoprotein B^15^. Transfection of PILRA enables the spreading of HSV-1 in normally resistant cell lines^15^. *PILRA* rs1859788 c.232A > G (p.Arg78Gly) is thought to be a functional variant in the region adjacent to its sialic binding pocket. This missense mutation (*PILRA* R78G), where glycine (G) coded by the G allele is substituted for arginine (R) coded by the A allele, is suggested to be a protective variant for AD^11^. The A allele of *PILRA* R78G attenuates infection through reduced binding for several of its ligands, including HSV-1 glycoprotein B^11^.

Environmental exposure to infectious pathogens like HSV-1 might contribute to the pathogenesis of AD^19,20^. HSV-1 infection in mouse models and 3D brain organoids has been shown to induce typical features of AD^21,22^. Epidemiological observations of an association between HSV-1 infection and increased AD risk have provided further support for the link in humans^8–10,23–26^. Antiviral drugs given in the event of a recurrent herpes infection seem to reduce this risk according to recent retrospective cohort studies^27–31^.

AD is probably a polygenic disorder involving multiple genes and their combined effects^32^. Different allelic combinations can explain, at least in part, why only a subset of those carrying HSV-1 develop AD, since the virus is highly prevalent^33^. The recent finding that the A allele of *PILRA* R78G might be a protective gene variant for AD needs to be further investigated^11^. The aim of this study was to ascertain if *PILRA* R78G was associated with the risk of subsequent AD independently, or, by modifying the effect of other known risk markers, such as *APOEε*4, *GM*17, and HSV-1, in a nested case–control study of 360 AD subjects and their matched controls from Northern Sweden Health and Disease Study (NSHDS). Also, the associations were validated using two independent whole-genome sequencing datasets from the National Institute of Mental Health (NIMH) and from the National Institute of Aging’s (NIA) Alzheimer’s disease Sequencing Project (ADSP): NIA ADSP.

## Results

The descriptive statistics of the 360 AD cases and 360 matched controls from the discovery dataset (i.e. NSHDS) are presented in Table 1. The mean time to event was 9.6 ± 4.1 years (i.e. time between blood collection and AD diagnosis). The mean age of AD diagnosis was 70.8 ± 6.4 years.

**Table 1:** Descriptive statistics in the discovery dataset, NSHDS.

	AD cases, n = 360	Controls, n = 360
Age at blood collection, y, mean ± SD	61.2 ± 5.6	61.2 ± 5.6
Age at diagnosis, y, mean ± SD	70.8 ± 6.4	
Sex, females, % (n)	75.3 (271)	75.3 (271)
MMSE at diagnosis, mean ± SD	21.9 ± 5.0	
*APOE* risk variants, % (n)^a^	61.3 (219)	24.4 (86)
*PILRA* R78G A/A, % (n)	6.0 (21)	7.6 (27)
*PILRA* R78G A/G, % (n)	38.6 (136)	37.9 (134)
*PILRA* R78G G/G, % (n)	55.4 (195)	54.5 (193)
*GM* 3/17	47.4 (166)	48.0 (169)
*GM* 17/17, % (n)	20.3 (71)	10.8 (38)
Anti-HSV-1 IgG + , % (n)	91.4 (329)	88.1 (317)
Anti-HSV IgG levels^b,c^	102.5 ± 21.4	102.5 ± 22.2
Anti-HSV IgM + , % (n)^c^	8.2 (27)	5.4 (17)

*AD* Alzheimer’s disease, *y* Years, *SD* Standard deviation, *n* Number, *MMSE* Mini-mental state examination, *APOE* Apolipoprotein E.

^a^Genotype ε3/ε4 or ε4/ε4.

^b^Expressed in arbitrary units.

^c^Among anti-HSV-1 IgG seropositive subjects.

**Figure 1:**
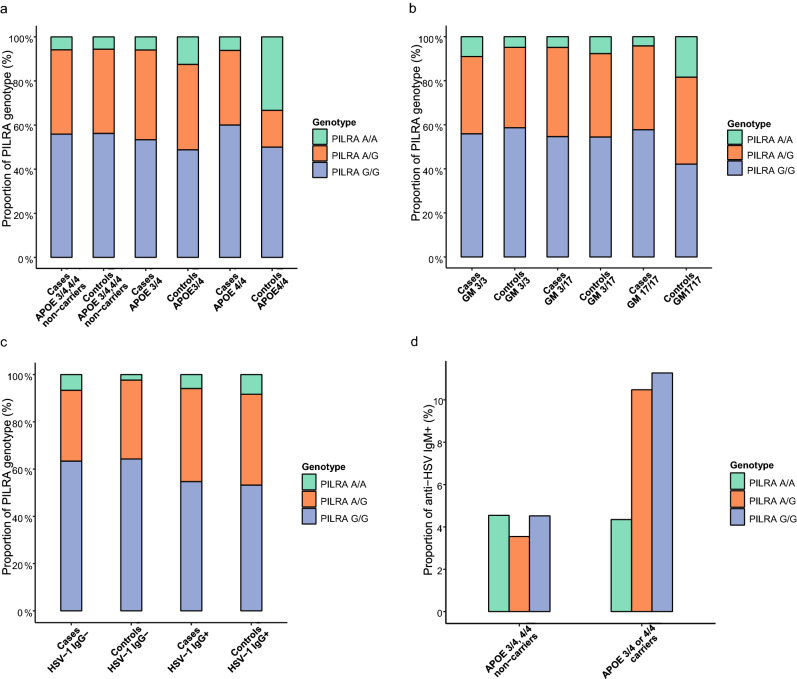
Proportions of *PILRA* R78G genotype and anti-HSV IgM + respectively. A) Stratified by *APOE*ε4 genotype and case–control status. B) Stratified by *GM* genotype and case–control status. C) Stratified by anti-HSV-1 IgG + and case–control status. D) Proportion of anti-HSV IgM + stratified by APOE risk variants and PILRA R78G genotype.

The *PILRA* R78-A allele was not associated with AD in the discovery dataset (crude Odds ratio (OR) 0.94, 95% confidence interval (CI) 0.74–1.21, *p* = 0.656; Table 2). The interactions terms were modelled using conditional logistic regression and additive coding for *PILRA* R78G-A and *GM*17 (see Methods). We found negative interactions between *PILRA* R78G-A x *GM*17 and *PILRA* R78G-A x *APOE* risk variants (*ε*3/ε4 or *ε*4/ε4) for the risk of AD (OR for the interaction 0.72, 95% CI 0.52–1.00 and 0.56, 95% CI 0.32–0.98 respectively; Table 3). The interaction term of *PILRA* R78G-A x anti-HSV-1 IgG seropositivity was not significant (Table 3). These interaction effects are also visualized in Fig. 1A–C where *PILRA* R78G is plotted against *APOE, GM* genotypes, and anti-HSV-1 IgG in separate groups.

**Table 2:** Conditional logistic regression of Alzheimer’s disease risk with the *PILRA* R78G-A allele, *APOE* risk variants, the *GM*17 allele and anti-HSV-1 IgG.

	OR	95% CI	*p*
*PILRA*-A	0.94	0.74–1.21	.656
*APOE* risk variants^a^	5.19	3.53–7.63	< .001
*GM*17	1.49	1.19–1.87	< .001
Anti-HSV-1 IgG +	1.44	0.88–2.36	.142

*OR* Odds ratio, *CI* Confidence interval, *APOE* Apolipoprotein E.

^a^Genotype ε3/ε4 or ε4/ε4.

**Table 3:** Conditional logistic regression of Alzheimer’s disease risk with interactions of *PILRA* R78G-A, *APOE* risk variants, *GM* 17/17 and anti-HSV-1 IgG +.

Variables	Model^a^		Model 2^b^		Model 3^c^	
	OR (95% CI)	*p*	OR (95% CI)	*p*	OR (95% CI)	*p*
*PILRA*-A	1.20 (0.82–1.75)	.346	1.24 (0.86–1.80)	.253	1.14 (0.52–2.51)	.743
*APOE* risk variants ^d^	7.17 (4.24–12.12)	< .001				
*PILRA*-A x *APOE* risk variants	0.56 (0.32–0.98)	.042				
*GM*17			1.78 (1.33–2.37)	< .001		
*PILRA*-A x *GM*17			0.72 (0.52–1.00)	.049		
Anti-HSV-1 IgG +					1.62 (0.88–2.97)	.118
*PILRA*-A x anti-HSV-1 IgG +					0.79 (0.35–1.83)	.592

*OR* Odds ratio, *CI* Confidence interval, *APOE* apolipoprotein E.

^a^Interaction model: *PILRA* R78G-A x *APOE* risk variants.

^b^Interaction model: *PILRA* R78G-A x *GM*17.

^c^Interaction model: *PILRA* R78G-A x anti-HSV-1 IgG +.

^d^Genotype ε3/ε4 or ε4/ε4.

Table 4 shows the descriptive statistics of subjects with different *PILRA* R78G genotypes among cases and controls separately. The distribution of *PILRA* R78G genotype in cases and controls, stratified by *APOE, GM*17, and anti-HSV-1 IgG status is also presented in Fig. 1A–C. Controls with *APOE* risk variants, the *GM*17 allele and anti-HSV-1 IgG antibodies all seemed to have higher frequencies of *PILRA* A/A genotype compared to their cases (Fig. 1A–C). In contrast, subjects (cases and controls combined) carrying both *PILRA* R78G A/A and *APOE* risk variants had lower frequencies of detectable anti-HSV IgM antibodies compared to subjects with *APOE* risk variants and non-*PILRA* R78G A/A genotypes (Fig. 1D).

**Table 4:** Descriptive statistics of *PILRA* R78G-A carriers and non-carriers stratified by case–control status.

	AD cases			Controls		
	*PILRA* A/A n = 21	*PILRA* A/G n = 136	*PILRA* G/G n = 195	*PILRA* A/A n = 27	*PILRA* A/G n = 134	*PILRA* G/G n = 193
Age at blood collection, y, mean ± SD	61.6 ± 5.1	61.3 ± 6.1	61.2 ± 5.2	59.4 ± 5.1	61.4 ± 5.7	61.3 ± 5.6
Age at diagnosis, y, mean ± SD	71.9 ± 6.2	72.0 ± 6.2	71.1 ± 6.0			
Sex, female, %, (n)	81.0 (17)	76.5 (104)	74.4 (145)	74.1 (20)	73.9 (99)	76.7 (148)
*APOE* risk variants, % (n)^a^	61.9 (13)	61.5 (83)	61.0 (119)	44.4 (12)	23.9 (32)	21.9 (42)
*APOEε*3/*ε*4	42.9 (9)	45.2 (61)	41.0 (80)	37.0 (10)	23.1 (31)	20.3 (39)
*APOEε*4/*ε*4	19.0 (4)	16.3 (22)	20.0 (39)	7.4 (2)	0.7 (1)	1.6 (3)
*GM 3/17*	38.1 (8)	50.0 (66)	46.4 (89)	48.1 (13)	48.5 (64)	47.7 (92)
*GM* 17/17, % (n)	14.3 (3)	20.5 (27)	21.4 (41)	25.9 (7)	11.4 (15)	8.3 (16)
Anti-HSV-1 IgG + , % (n)	90.5 (19)	93.4 (127)	90.3 (176)	96.3 (26)	89.6 (120)	86.0 (166)
Anti-HSV IgG levels^b,c^	106.4 ± 16.6	102.0 ± 23.2	102.3 ± 20.8	99.4 ± 21.2	99.8 ± 23.6	105.3 ± 20.7
Anti-HSV IgM + , % (n)^c^	10.5 (2)	8.7 (11)	7.4 (13)	0 (0)	4.2 (5)	7.2 (12)

*AD* Alzheimer’s disease, *y* years, *SD* Standard deviation, *n* number, *APOE* apolipoprotein E.

^a^Genotype ε3/ε4 or ε4/ε4.

^b^Expressed in arbitrary units.

^c^Among anti-HSV-1 IgG seropositive subjects).

Next, we sought to assess the main or interaction effects of *PILRA* R78G in two AD whole-genome sequencing datasets with different study designs: a large family-based AD sample from NIMH and an AD case–control dataset from NIA ADSP (Table 5). The case–control sample from the NIA ADSP contained three subcohorts: a Non-Hispanic White cohort, an African-American cohort and a Hispanic cohort.

**Table 5:** Description of WGS datasets.

	NIMH, family-based		NIA ADSP unrelated, non-Hispanic whites	
	AD cases, n = 966	Controls, n = 427	AD cases, n = 983	Controls, n = 686
Age at onset or last exam, y, mean ± sd	71.9 ± 8.4	72.9 ± 12.2	74.9 ± 8.9	78.9 ± 6.6
Sex, females, % (n)	72.5 (700)	58.1 (248)	44.9 (441)	57.7 (396)
*APOE* risk variants, % (n) ^a^	68.4 (661)	47.8 (204)	50.4 (495)	21.6 (148)
*PILRA* R78G A/A, % (n)	8.6 (83)	10.8 (46)	9.8 (96)	9.9 (68)
*PILRA* R78G A/G, % (n)	37.3 (360)	41.2 (176)	40.8 (401)	41.5 (285)
*PILRA* R78G G/G, % (n)	54.1 (523)	48.0 (205)	49.4 (486)	48.5 (333)
*GM* 17/17, % (n)	15.6 (151)	15.0 (64)		

*AD* Alzheimer’s disease, *y* Years, *SD* Standard deviation, *n* Number, *NIMH* National Institute of Mental Health, *NIA* National Institute of Ageing, *ADSP* Alzheimer’s Disease Sequencing Project.

^a^Genotype ε3/ε4 or ε4/ε4.

Using transmission family-based approaches, we saw an association of AD risk with *PILRA* R78G (*p* = 0.0495) and *APOE* rs429358 (ε4, *p* = 1.78 × 10^−15^) and rs7412 (ε2, *p* = 5.01 × 10^−5^) SNPs, but not with *GM*17 (rs1071803, *p* = 0.9). This method is used to evaluate both linkage and association with the phenotype of interest in family pedigrees. When including one of the following interaction terms: *PILRA* R78G × *APOE* risk variants or *PILRA* R78G × *GM* 17/17, we found that the family-based joint test for the main effect *PILRA* G78R and the interaction effect *PILRA* R78G × *GM* 17/17 was significant (*p* = 0.02, Table 6). However, none of the interaction terms in each of the two models was significant. Finally, in the non-Hispanic white subpopulation of the NIA ADSP dataset (n = 1669), *PILRA* R78G was not associated with AD (*p* = 0.94). The variant rs1071803, which codes for *GM*17, was missing in NIA ADSP and the interaction term *PILRA* R78G × *APOE* risk variants were not significant (*p* = 0.66 using additive coding and *p* = 0.27 using recessive coding).

**Table 6:** Family-based association tests (additive model) in the NIMH cohort for main, interaction and joint effects.

rsid	interaction_term	Minor allele frequency	main effect p-value	interaction effect p-value	joint p-value
rs1859788	ε3/ε4 or ε4/ε4	0.2860	0.0495	0.2294	0.0787
rs1859788	*GM* 17/17	0.2860	0.0495	0.3224	0.0205

Since the FBAT test statistics are derived based on a score test approach, no OR is estimated.

## Discussion

The key finding of our study is that the *PILRA* R78G-A allele negatively modifies the effect of *APOE* and *GM*17 high-risk variants on AD risk (OR for the *GM*17 interaction 0.72, 95% CI 0.52–1.00 and OR for the *APOE* interaction 0.56, 95% CI 0.31–0.98; Table 3 in the discovery cohort). The effect modification seems to be of increased strength in *APOEε*4 and *GM*17 homozygotes (Fig. 1A, B), revealing a potential dose-dependent pattern. Similarly, we found a significant joint effect of *PILRA* R78G and *PILRA* R78G × *GM* 17/17 for AD in the replication cohort. While having the *PILRA* R78G-A allele was associated with reduced risk of AD in the family cohort, this association was not replicated in the other two samples.

Previous epidemiological studies have shown that HSV-1 is associated with increased AD risk in genetically predisposed individuals carrying the *APOEε*4 allele or other AD risk genes^7–10,23^. The finding that the *PILRA* R78G-A allele might modify the risk of AD in *APOEε*4 and *GM*17 carriers (Table 3) might further enhance our understanding of the complex gene-gene and gene-environment interactions for HSV1-associated AD risk.

*PILRA* R78G has previously been linked to both HSV-1 and AD^11,15^. The A allele of *PILRA* R78G causes a conformational change in its sialic binding pocket, which leads to impaired binding capacity for HSV-1 and other ligands^11^. This could make target cells less susceptible to HSV-1 infection through reduced HSV-1 cell fusion, and limit viral entry into neurons in the brain, thus offering some protection against HSV-1-associated AD. The effect of *PILRA* could also possibly be explained by fewer latently infected neurons in the periphery, which correlate with lower reactivation rates of HSV-1^34^. Importantly, PILRA also function as an inhibitory regulator of microglia activation^35^, and reduced PILRA signaling in R78G-A allelic variants could result in the enhancement of microglial activity^11^. It is therefore possible that the decrease in AD risk associated with having the *PILRA* R78G-A allele might be attributed to more properly regulated microglia and possibly improved amyloid-β clearance^36^. However, the exact role of microglia in AD initiation and progression remains to be fully elucidated, and it might vary during the course of the disease.

In the discovery cohort, we observed a potential modifying effect of *PILRA* R78G A/A on the risk of having anti-HSV IgM antibodies (a marker of recent HSV reactivation) among carriers of *APOE* risk variants (Fig. 1D). Notably, we have previously shown that having *APOE* risk variants were associated with a higher prevalence of anti-HSV IgM antibodies in the NSHDS sample^6^, thus an association that seems to be negatively modified by *PILRA*. Figure 1C illustrates that *PILRA* R78G A/A homozygosity also could have a protective impact on the HSV-1 associated AD risk, although not statistically significant (Table 3). Herein, HSV-1 seropositive controls had a higher frequency of *PILRA* R78G A/A genotypes compared to HSV-1 seronegative controls.

The primary strength of this study is that controls, sampled from the same population, were closely matched on possible confounding and demographic variables. Another major strength is the prospective design, where blood specimens were obtained several years prior to the disease onset, making it possible to estimate future disease risk. Limitations include the observational nature of our study, as potential unaccounted confounding factors could influence the associations and that the AD diagnoses were clinical and not based on evidence of amyloid deposition or pathologic tau. A further limitation noticed was that only 5.3% of AD cases and 7.3% of controls were *PILRA* R78G A/A homozygotes (Table 1), suggesting that this genotype is not common in the studied population. The allele frequency of *PILRA* rs1859788 seems to vary globally, and is higher in the East Asian population^37^. This variation in allele frequency could possibly explain the lack of association between AD and *PILRA* R78G in the NSHDS and NIA ADSP material, which was indicated by another study^11^ and the family-based NIMH dataset.

## Conclusion

Here, we report a negative effect modification by the *PILRA* R78G-A allele on *APOE* and *GM*17 risk variants for future AD risk in two independent datasets. This observation might provide further insight into the complex genetics of HSV1-associated AD.

## Methods

### Study design

#### Discovery dataset NSHDS

We used a nested case–control study design, where 360 subjects clinically diagnosed with AD were identified from the population-based Northern Sweden Health and Disease study (NSHDS)^38^. The NSHDS consists of three subcohorts: the Västerbotten Intervention Programme (VIP), the Mammography Screening Project (MA), and The Northern Sweden Monica Project (MO). Blood samples were previously drawn and stored in the Medical Biobank in Umeå, extracted for analysis on average 9.6 years before the AD diagnosis. Controls without neurodegenerative disorders were randomly selected from the NSHDS cohort and matched 1:1 by age, sampling dates, sex, and subcohort. The diagnostic procedure and selection of subjects have been described in a previous publication^25^.

#### NIMH family-based dataset and ADSP case–control dataset

The results were cross-validated using two independent whole-genome sequencing datasets, a family-based AD cohort from NIMH and an AD case–control sample from the NIA (ADSP).

#### Genotyping in NSHDS

Samples were genotyped for *APOE* (rs429358 and rs7412) and *PILRA* R78G (rs1859788) using Illumina genome-wide array Human-OmniExpress24 (deCODE genetics, Reykjavik, Iceland)^9^. QPCR-based genotyping assays^11,39^ were employed for confirmation of inconclusive sequences. A custom design TaqMan genotyping assay was employed for genotyping of the *GM*3 and17 alleles (i.e. to determine *GM* 3/3, *GM* 3/17 and *GM* 17/17 genotypes)^6^.

#### WGS analysis in NIMH and ADSP

Whole genome sequencing in the National Institute of Mental Health (NIMH) AD cohort and AD diagnoses are described elsewhere^40,41^. Variant calls in vcf format for the National Institute of Aging’s (NIA) Alzheimer’s disease sequencing project (ADSP) cohort were obtained from the National Institute on Aging Genetics of Alzheimer’s Disease Data Storage Site (NIAGADS) under accession number: NG00067. The NIA ADSP dataset was divided into three subcohorts: Non-Hispanic White, African-American and Hispanic based on derived principal components. In order to derive more recent admixture principal components were calculated based on 100,000 rare variants using a modified genetic relationship matrix based on the Jaccard index^42^. Outliers based on principal components were excluded.

#### Serology—NSHDS

Enzyme-linked immunosorbent assays were used for the detection of anti-HSV IgG, anti-HSV-1 IgG, and anti-HSV IgM as previously described^25^.

### Statistical analyses

#### Variables for APOE, GM, and PILRA R78G genotypes

We used additive coding for *GM*17 and PILRA R78G, as having 0, 1 or 2 copies of the minor allele (i.e. the *PILRA* R78G-A or *GM*17 alleles). The *APOE* variable was dichotomized as having high risk variants (ε3/ε4 or ε4/ε4) compared to ε3/ε4 and ε4/ε4 non-carriers. The rationale for dichotomizing *APOE* is that the effect of *APOE*ε4 on AD risk is not additive, and the *APOE* locus is not bi-allelic.

#### APOE, GM, PILRA R78G, HSV-1, and the risk of AD in NSHDS

Associations between the risk of AD and the *PILRA* R78G-A allele were assessed by conditional logistic regression models. Interaction models were fitted for *PILRA* R78G-A and AD with interaction terms for *PILRA* R78G-A x *APOE* risk variants, *PILRA* R78G-A x *GM*17 and *PILRA* R78G-A x anti-HSV-1 IgG seropositivity. Each interaction term was modeled separately to estimate the effect modification by the *PILRA* R78G-A allele on AD risk per these factors.

The gene variables contained missing data ranging from n = 3 to 10 (*APOE:* n = 3 cases and n = 7 controls, PILRA R78G: n = 8 cases and n = 6 controls, *GM:* n = 10 cases and n = 8 controls). Subjects with missing values were omitted from the statistical analyses. This strategy was chosen since data can be assumed to be missing completely at random due to their blood samples containing insufficient amounts of DNA.

Statistical analyses were performed using R version 4.1.3. A two-tailed *p*-value < 0.05 was considered significant. The codes are available as supplementary files (Supplementary file 1: discovery cohort and Supplementary file 2: replication cohorts).

#### APOE, GM, PILRA R78G, and the risk of AD in NIMH and ADSP

PLINK2^43^ (www.cog-genomics.org/plink/2.0/) was used to pre-process and extract variants of interest. In the NIMH cohort, we used a robust gene-by-environment test^44^, which is based on the family-based association test (FBAT)^45^, a generalization of the transmission disequilibrium test. We used the function “fbatge” from the “fbati” package in R. In the case–control cohort, we used PLINK2 and R to perform logistic regression with covariates (Age, Sex, Sequencing center, and first 5 principal components to adjust for the population structure) and the corresponding interaction term. If not mentioned otherwise, we considered an additive model for *PILRA* G78R and considered the following interaction terms: *PILRA* R78G A/A x *APOE* risk variants, *PILRA* R78G A/A x *GM* 17/17, *PILRA* R78G A/A x *APOE,* or *GM* 17 risk variants. Information on anti-HSV-1 IgG seropositivity was not available in WGS cohorts.

### Ethical approval

The study was performed in accordance with the Declaration of Helsinki and was approved by the Regional Ethical Review Board in Umeå, Sweden (diary no. 09-190 M and 2017/18-31). All participants provided informed consent for long-term storage of blood specimens and for research on the stored samples.

## Supplementary Information

Supplementary Information 1.

Supplementary Information 2.

## Data Availability

Discovery cohort, NSHDS: The dataset generated and analyzed during the current study is uploaded as a supplementary file. Additional information is available from the authors upon reasonable request, and after review and with permission from The Biobank Research Unit at Umeå University. Replication cohorts: The NIMH WGS dataset analyzed during the current study was funded by Cure Alzheimer's Fund, a non-profit organization, and is available from the authors on reasonable request. The NIA ADSP WGS dataset is available from DSS NIAGADS (https://dss.niagads.org/) under accession number: NG00067. Data used in preparation of this article were in part obtained from the Alzheimer’s Disease Neuroimaging Initiative (ADNI) database (adni.loni.usc.edu). As such, the investigators within the ADNI contributed to the design and implementation of ADNI and/or provided data but did not participate in analysis or writing of this report. A complete listing of ADNI investigators can be found at: http://adni.loni.usc.edu/wp-content/uploads/how_to_apply/ADNI_Acknowledgement_List.pdf.
