# Supplementary material for: PILRA polymorphism modifies the effect of APOE4 and GM17 on Alzheimer’s disease risk
Source: Sci Rep. 2022 Aug 2;12:13264. doi: 10.1038/s41598-022-17058-6 (PMC9346002; doi:10.1038/s41598-022-17058-6)
Supplement: Supplementary file 1 — Supplementary Information 1. [file 41598_2022_17058_MOESM1_ESM.pdf]

```
View (PILRA_df)

# additive coding for GM17

PILRA_df$GM17add <- PILRA_df$GM317

PILRA_df$GM17add [PILRA_df$GM317 == 33] <- 0

PILRA_df$GM17add [PILRA_df$GM317 == 317] <- 1

PILRA_df$GM17add [PILRA_df$GM317 == 1717] <- 2

table(PILRA_df$GM17add)

PILRA_df$GM17add <- as.numeric(PILRA_df$GM17add)


# additive coding for PILRA

PILRA_df$PILRAadd <- PILRA_df$PILRA

PILRA_df$PILRAadd [PILRA_df$PILRA == "AG"] <- 1

PILRA_df$PILRAadd [PILRA_df$PILRA == "AA"] <- 2

PILRA_df$PILRAadd [PILRA_df$PILRA == "GG"] <- 0

table(PILRA_df$PILRAadd)

PILRA_df$PILRAadd <- as.numeric(PILRA_df$PILRAadd)


# dichotomous coding for APOE as risk variants vs non-risk variants

PILRA_df$APOErv <- PILRA_df$APOE

PILRA_df$APOErv [PILRA_df$APOE == 34 |PILRA_df$APOE == 44 ] <- 1

PILRA_df$APOErv [PILRA_df$APOE == 22 |PILRA_df$APOE == 23 | PILRA_df$APOE
== 24 | PILRA_df$APOE == 33 ] <- 0

table(PILRA_df$APOErv)

PILRA_df$APOErv <- as.factor(PILRA_df$APOErv)


# get required package

library(survival)


# fit model PILRA


clogitPILRA <- clogit(case_control ~ PILRAadd

                      + strata (case_set), data = PILRA_df)
```

```
summary(clogitPILRA)


# fit interaction model PILRA:APOE


clogitAPOE <- clogit(case_control ~ APOErv * PILRAadd
                     + strata (case_set), data = PILRA_df)
summary(clogitAPOE)


# fit interaction model PILRA:GM17


clogitGM <- clogit(case_control ~ GM17add * PILRAadd
                     + strata (case_set), data = PILRA_df)
summary(clogitGM)

# fit interaction model PILRA:HSV-1

PILRA_df$HSV1IgGpos <- as.factor(PILRA_df$HSV1IgGpos)

clogitHSV1 <- clogit(case_control ~ HSV1IgGpos * PILRAadd
                   + strata (case_set), data = PILRA_df)
summary(clogitHSV1)
```
