# Supplementary material for: PILRA polymorphism modifies the effect of APOE4 and GM17 on Alzheimer’s disease risk
Source: Sci Rep. 2022 Aug 2;12:13264. doi: 10.1038/s41598-022-17058-6 (PMC9346002; doi:10.1038/s41598-022-17058-6)
Supplement: Supplementary file 2 — Supplementary Information 2. [file 41598_2022_17058_MOESM2_ESM.pdf]

```
library(data.table)
library(dplyr)
library(tidyr)
library(fbati)

#FBAT interaction analysis
ped<-fread.ped("temp.ped")
phe<-fread.phe("temp.phe")

tab1<-fbatgeAll(ped,phe,env="has_e4")
tab2<-fbatgeAll(ped,phe,env="gm17_17")

final<-filter(tab1,Marker=="X7.100374211.a.g") %>%
  mutate(rsid="rs1859788",interaction_term="has_e4") %>%
  select(rsid,interaction_term,everything()) %>% select(-Marker) %>%
  rbind(filter(tab2,Marker=="X7.100374211.a.g") %>%
          mutate(rsid="rs1859788",interaction_term="gm17_17") %>%
          select(rsid,interaction_term,everything()) %>% select(-Marker))

fwrite(final,file="results_table6.txt",quote=F,row.names=F,sep='\t')

# Case-control interaction analysis
geno<-fread("geno_snps.raw",data.table=F) %>%
  mutate(has_e4=case_when((rs429358==1 & rs7412==0) | (rs429358==2 &
rs7412==0) ~ 1,
                          is.na(rs429358) | is.na(rs7412) ~ NA_real_,
                          TRUE ~ 0),
         PILRA_rec=case_when(rs1859788_A==2 ~ 1,
                             is.na(rs1859788_A) ~ NA_real_,
                             TRUE ~ 0),
         IID=as.character(IID))
pheno<-fread("pheno.txt",data.table=F) %>%
```

```
  left_join(geno)


#additive coding

fit<-glm(Affection.Status~SEQ_CENTER+Age+Sex+PC1+PC2+PC3+PC4+PC5+rs1859788_A*has_e4,family = binomial(link = "logit"),data=pheno)

summary(fit)

#recessive coding

fit<-glm(Affection.Status~SEQ_CENTER+Age+Sex+PC1+PC2+PC3+PC4+PC5+PILRA_rec*has_e4,family = binomial(link = "logit"),data=pheno)

summary(fit)
```
